# The Influence of Methyl Jasmonate on Expression Patterns of Rosmarinic Acid Biosynthesis Genes, and Phenolic Compounds in Different Species of *Salvia* subg. *Perovskia* Kar L.

**DOI:** 10.3390/genes14040871

**Published:** 2023-04-05

**Authors:** Farzad Kianersi, Davood Amin Azarm, Farzaneh Fatemi, Bita Jamshidi, Alireza Pour-Aboughadareh, Tibor Janda

**Affiliations:** 1 School of Environmental Sciences, University of Guelph, 50 Stone Road East, Guelph, ON N1G 2W1, Canada; 2 Department of Horticulture Crop Research, Isfahan Agricultural and Natural Resources Research and Education Center, AREEO, Isfahan P.O. Box 81785-199, Iran; 3 Department of Agronomy and Plant Breeding, Faculty of Agriculture, Bu-Ali Sina University, Hamedan P.O. Box 6517838695, Iran; 4 Department of Food Security and Public Health, Khabat Technical Institute, Erbil Polytechnic University, Erbil 44001, Iraq; 5 Seed and Plant Improvement Institute, Agricultural Research, Education and Extension Organization (AREEO), Karaj P.O. Box 3158854119, Iran; a.poraboghadareh@edu.ikiu.ac.ir; 6 Department of Plant Physiology and Metabolomics, Agricultural Institute, Centre for Agricultural Research, 2462 Martonvásár, Hungary

**Keywords:** *Salvia*, methyl jasmonate, total phenolic, total flavonoid content, transcriptome

## Abstract

*Salvia yangii* B.T. Drew and *Salvia abrotanoides* Kar are two important fragrant and medicinal plants that belong to the subgenus *Perovskia*. These plants have therapeutic benefits due to their high rosmarinic acid (RA) content. However, the molecular mechanisms behind RA generation in two species of *Salvia* plants are still poorly understood. As a first report, the objectives of the present research were to determine the effects of methyl jasmonate (MeJA) on the rosmarinic acid (RA), total flavonoid and phenolic contents (TFC and TPC), and changes in the expression of key genes involved in their biosynthesis (*phenylalanine ammonia lyase* (*PAL*), *4-coumarate-CoA ligase* (*4CL*), and *rosmarinic acid synthase* (*RAS*)). The results of High-performance liquid chromatography (HPLC) analysis indicated that MeJA significantly increased RA content in *S. yungii* and *S. abrotanoides* species (to 82 and 67 mg/g DW, respectively) by 1.66- and 1.54-fold compared with untreated plants. After 24 h, leaves of *Salvia yangii* and *Salvia abrotanoides* species treated with 150 M MeJA had the greatest TPC and TFC (80 and 42 mg TAE/g DW, and 28.11 and 15.14 mg QUE/g DW, respectively), which was in line with the patterns of gene expression investigated. Our findings showed that MeJA dosages considerably enhanced the RA, TPC, and TFC contents in both species compared with the control treatment. Since increased numbers of transcripts for PAL, 4CL, and RAS were also detected, the effects of MeJA are probably caused by the activation of genes involved in the phenylpropanoid pathway.

## 1. Introduction

Medicinal and aromatic plants are used in pharmaceuticals, cosmetics, personal care items, incense, and food to treat diseases, maintain health, and prevent them [1]. These plants are becoming more popular as public interest in natural resources increases daily [2,3].

One of the most significant genera in the *Lamiaceae* family, *Salvia* (garden sage), has around 1000 species worldwide [4]. Numerous *Salvia* species are used to make herbal tea, as well as in cosmetics, fragrances, and medicines [5]. The biological properties of plants in this genus, such as antioxidant, anti-tumor, anti-inflammatory, antibacterial, antidiabetic, and anxiolytic characteristics, further set them apart [6,7,8,9].

Several perennial, shrubby, and fragrant species with possible medical uses may be found in the subgenus *Perovskia* Kar of the *Salvia* expanded genus [10]. *Salvia yangii* B.T. Drew (formerly *Perovskia atriplicifolia* Benth.) and *Salvia abrotanoides* Karel (formerly known as *Perovskia abrotanoides* Kar) are the two most common among them, being found in extensive regions including the western regions of Iran, Pakistan, Tibet, and Xinjiang in China [11,12,13,14].

Both *S. abrotanoides* and *S. yangii* are used as traditional medicines in the regions where they grow naturally. Diabetes, diarrhea, scabies, fever, wound healing, and antibiotic therapy are among the conditions that *S. yangii* is used to treat [15,16,17,18,19]. *S. yangii* has been described as a potent analgesic and parasiticide in traditional Tibetan and Chinese medicine [20,21]. Locally, *S. abrotanoides*, also known as “Brazambol” in common parlance, is used as a sedative, analgesic, and antiseptic as well as a therapy for toothache, typhoid, fever, headache, cardiovascular disorders, gonorrhoea, vomiting, liver fibrosis, painful urination, and cough [22,23,24,25]. Moreover, *S. abrotanoides* was shown to possess cytotoxic, anti-plasmodial, and anti-inflammatory pharmacological activities, all of which have been employed in Iranian traditional medicine to cure leishmaniasis [26,27].

**Figure 1:**
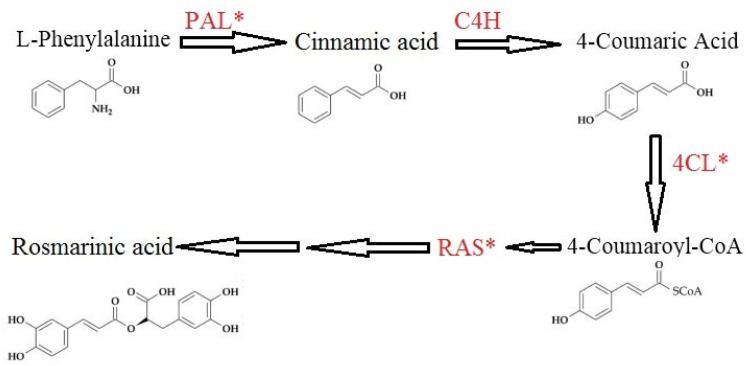
The biosynthesis of rosmarinic acid. The enzyme genes for *phenylalanine ammonia lyase* (*PAL*), *4-coumarate-CoA ligase* (*4CL*), and *rosmarinic acid synthase* (*RAS*), among others, are marked with an asterisk.

Rosmarinic acid (RA) has been found to be the main phenylpropanoid present in the medicinal plant *Slavia* [28]. This molecule has many roles, such as antioxidant, anti-inflammatory, and antibacterial activities [29,30,31,32]. The main active phenolic compound, RA, chemically, is an ester of 3,4-dihydroxyphenyllactic acid and caffeic acid (Figure 1). It is a substance that occurs naturally and is present in many medicinal plants [33]. RA is found to be an active component in a number of *Lamiaceae*-related medicinal plants, but it may also be found in plants from other floras [34,35].

The RA biosynthesis pathway genes in two species of *Salvia* subgenus Perovskia (*S. yangii* and *S. abrotanoides*) treated with MeJA have not yet been assessed, despite the fact that their expression has been investigated in a range of plant species. Production and accumulation of these molecules are influenced by various time scales, developmental phases, and responses to biotic and abiotic stimuli [36,37,38,39]. Elicitors are one of the important factors that trigger plant defense mechanisms and cause the accumulation of certain secondary metabolites [40,41,42]. Methyl jasmonate (MeJA) as a signaling molecule is essential for the signal transduction pathway. Exogenous application of this potent abiotic elicitor in plants is employed to encourage the production of the desired secondary compounds [43,44]. Several medicinal plants, such as *Salvia miltiorrhiza Bunge* [45], *Satureja khuzistanica Jamzad* [46], *Capparis spinosa* L. [47,48], *Thymus migricus Klokov and Des.-Shost* [49], *Melissa officinalis* L. [50], *Foeniculum vulgare* Mill. [51], and *Coriandrum sativum* L. [52], have been the subject of extensive MeJA research into the regulation of secondary metabolism. Numerous studies [53,54,55] have shown that MeJA has a positive influence on RA levels and the expression levels of related essential genes in plants.

Despite the fact that many studies have looked into the effects of elicitors, such as MeJA, on the formation of phenolic acids, we are unaware of any research that has looked into RA and gene expression patterns in RA-related genes in *S. yangii* and *S. abrotanoides* species. Hence, the main objective of the present study was to investigate changes in RA-related gene expression (PAL, 4CL, and RAS), phenolic compound accumulation, and RA concentration in two species of *Salvia* (*S. yangii* and *S. abrotanoides*) in response to the MeJA treatment. Furthermore, we looked for an association between the expression patterns of genes involved in the RA biosynthesis pathway and the RA content in the leaves in two species of *Salvia* during the vegetative growth stage after the MeJA treatment.

## 2. Result and Discussion

### 2.1. RA Changes at Different MeJA Concentrations

**Figure 2:**
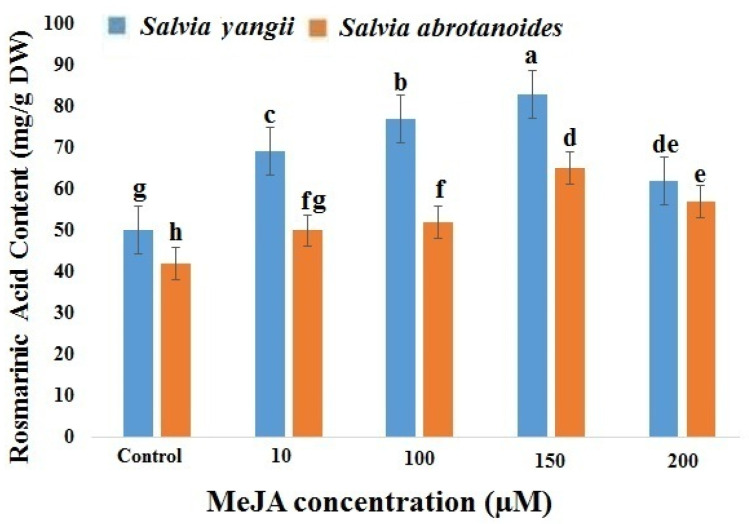
Concentration of rosmarinic acid in two species of *Salvia* (subgenus *Perovskia* Kar). Duncan’s test claims that bars labeled differently reflect statistical significance at the 1% level. Error bars represent the standard deviation of the data. Means followed by the same letters in each column are not significantly different (*p* < 0.01).

As shown in Figure 2, the different MeJA treatments significantly affected RA, a key component of two *Salvia* species. Additionally, Figure 2 illustrates how, after 24 h, the RA content in both treated *Salvia* species dramatically increased under the MeJA elicitor. In general, RA accumulation was greater in the *S. yangii* than the *S. abrotanoides* at all MeJA concentrations. In the *S. yangii* species, the concentration of RA increased by 1.24, 1.38, 1.66, and 1.54 times greater than in untreated plants when they were exposed to 10, 100, 150, and 200 µM MeJA, respectively. The RA quantity in the *S. abrotanoides* species also increased in accordance with the application of various MeJA treatments and was 1.19, 1.23, 1.54, and 1.35 times more than their control (Figure 2). According to the results, the RA values of two *Salvia* species were different, which indicates that RA production is affected by genotype-specific responses to stimuli in abiotic stresses.

Our results imply that MeJA treatments may increase the amount of RA in various *Salvia* species, which in turn has an effect on the gene expression patterns of genes implicated in RA synthesis. On the other hand, our findings support those of earlier research [47,48,49,56] and suggest that *Salvia* plants’ RA levels vary depending on their genotype and dose. It has been reported that the treatments with 100–150 µM MeJA result in the accumulation of high levels of RA in *Lamiaceae* families [50,57]. Moreover, treating caper plants with 150 µM MeJA might stimulate the production of flavonoids [20,58]. According to some studies, MeJA stimulates the PAL enzyme’s activity, which may contribute to the growth of RA in diverse plants [46,54]. In fact, these results imply that MeJA could affect the increase in RA amount under 100- to 150-M therapy [50]. According to our findings, both ecotypes’ metabolic characteristics were noticeably impacted by the MeJA that was delivered. Moreover, the increase in RA accumulation through the MeJA may be due to the stimulation of biosynthetic pathways and the activation of related genes to induce radical scavenging through phenolic components. Both species saw a rise in RA after MeJA, although the *S. yangii* species experienced this increase more quickly.

Previous studies corroborate our findings about the effect of MeJA at varying doses on RA buildup and gene expression in *Salvia* spp. *Satureja khuzistanica* nodal cultures, *Agastache rugosa* Kuntze cell cultures, and *Coleus forskohlii* hairy root cultures all showed that MeJA is the most efficient elicitor for the development of RA, supporting this concept [57,58,59]. Park et al. [60] reported that both abiotic and biotic elicitors stimulated RA production in *A. rugosa* callus suspension cultures. A previous study conducted by Kintzios et al. [61] showed that cell suspension cultures increased RA at a rate of up to 10 mg/g dry weight in *Ocimum basilicum*. After MeJA treatment, *Lithospermum erythrorhizon* cells showed a 10-fold increase in RA content, which was in accordance with Mizukami et al. [62].

### 2.2. TPC and TFC as a Function of MeJA Concentration

**Figure 3:**
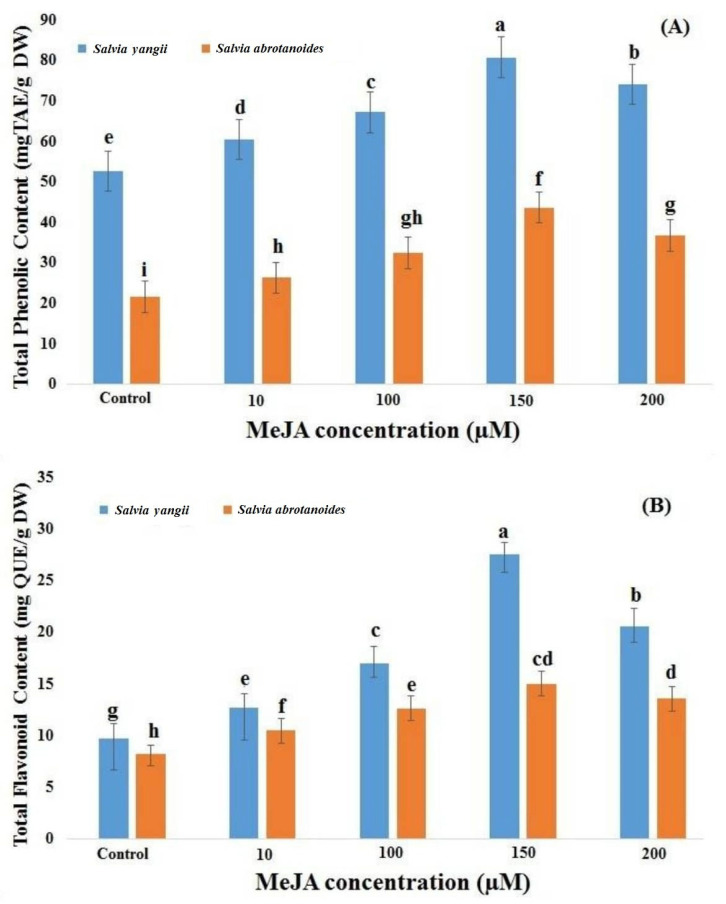
The effects of MeJA on TPC (**A**) and TFC (**B**) in two species of the genus *Salvia*, subgenus *Perovskia* Kar. Means and standard deviations (*n* = 3) are shown for the data. Duncan’s test claims that if there are different letters in each column, then it is statistically significant at the 1% level. Error bars depict the standard error values. Means followed by the same letters in each column are not significantly different (*p* < 0.01).

The TPC and TFC levels of two *Salvia* sub-genus *Perovskia* Kar leaves are obviously impacted by all concentrations of MeJA (Figure 3). It is probable that the pattern of RA content changes during the MeJA treatment was reflected in the pattern of TPC and TFC changes in both *S. yangii* and *S. abrotanoides* species (Figure 2 and Figure 3A,B). Additionally, as shown by our findings, MeJA had a distinct degree of influence on phenol and flavonoid accumulation in the *S. yangii* species as compared to the *S. abrotanoides* species. In accordance with previous studies, our results revealed that MeJA as a signaling molecule plays a key role in flavonoid formation [63,64]. Hence, these genes’ elevated expression in response to MeJA is almost certainly a result of the subsequent rise in phenolic compounds.

Concentrations of 10, 100, 150, and 200 µM MeJA raised the TPC in *S. yangii* by 1.14, 1.27, 1.53, and 1.41 times, respectively, compared to the untreated plants (Figure 3A). Accordingly, TPC values for the *S. abrotanoides* treated with the various doses of MeJA were tested and were, correspondingly, 1.21, 1.5, 2.02, and 1.7 times higher than the controls (Figure 3A). Moreover, *S. yangii* species treated with different doses of MeJA produced TFCs of up to 12.68, 17, 27.52, and 20.59 mg QUE/g DW, which were 1.30, 1.74, 2.82, and 2.11 times higher than the control plants, respectively (Figure 3B). TFC levels in the *S. abrotanoides* species were increased by MeJA treatments; these increases were about 1.27, 1.53, 2.07, and 1.64 times greater than the levels in untreated plants (Figure 3B). These results corroborate those of another study [65], which discovered species-specific differences in *Salvia* leaf TPC and TFC. In both ecotypes, TPC values were higher than TFC in both untreated and all treated leaves, supporting previous findings [66]. Recent investigations [67,68,69] agree with ours in showing that MeJA stimuli greatly influenced TPC in several plants. Researchers found that MeJA increased secondary metabolite accumulation in various plant species [70,71,72].

Higher quantities of polyphenolic compounds may come from the more rapid breakdown of larger phenolic compounds into smaller molecules, as shown by the research of Jaafar et al. [73]. The major regulating enzyme in phenylpropanoid metabolism, PAL, has been linked to the induction of TPC and TFC in two species of the subgenus *Perovskia* after treatment with MeJA [71]. Many plants’ expression of the phenylpropanoid biosynthesis genes (PAL, C4H, and 4CL) is inversely proportional to their flavonoid concentration [74,75]. Hence, the increased synthesis of RA and phenolic compounds after MeJA treatments was consistent with the expression of genes involved in the RA biosynthetic pathway.

### 2.3. MeJA’s Effects on the Expression of the PAL, 4CL, and RAS Genes

**Figure 4:**
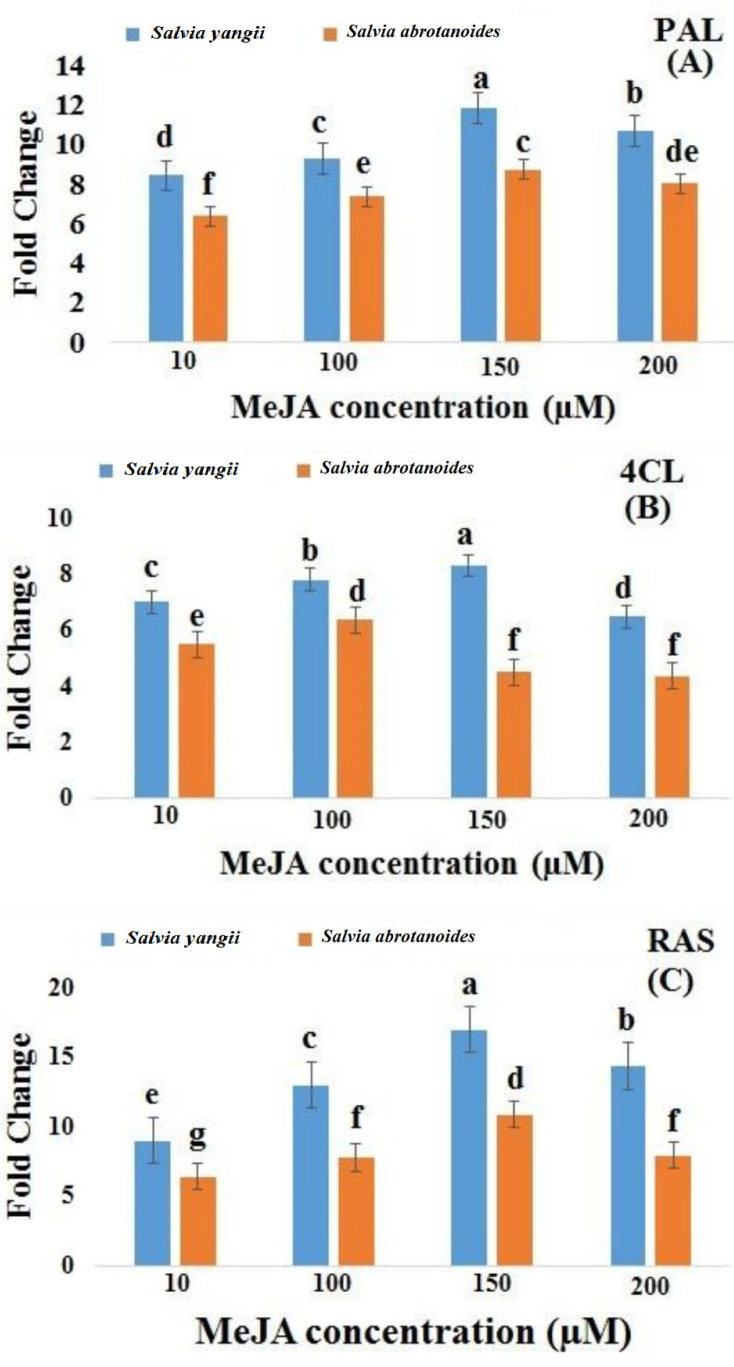
The relative expression of *PAL* (**A**), *4CL* (**B**), and *RAS* (**C**) genes in the control and MeJA-treated *Salvia* plants (fold-changed). Duncan’s test indicates a statistically significant (*p* ≤ 0.01) difference between bars labeled with different letters. Error bars depict the standard error values. Means followed by the same letters in each column are not significantly different (*p* < 0.01).

Changes in the transcript abundance of genes involved in the process that creates RA were investigated using real-time PCR, and the relationship between RA accumulation and gene expression in two species of *Salvia* that were subjected to varying dosages of MeJA was also analyzed (Figure 4). Results show that three genes investigated in *Salvia* plants had significantly different mRNA transcript levels in response to different MeJA treatments. It is important to note that MeJA-treated *S. yangii* had higher transcriptional levels of RA biosynthetic genes than MeJA-treated *S. abrotanoides*. Possible explanation for increased RA production in MeJA-treated plants, especially at the beginning and end of the RA biosynthetic pathway and during the expression of *PAL* and RAS.

Our results showed that the expression level of *PAL* in the *S. yangii* species enhanced from 8.42-fold at MeJA 10 M to 11.83-fold at MeJA 150 μM and remained almost the same at 10.67-fold at MeJA 200 μM (Figure 4A). At MeJA 10 M and 100 μM, the transcript level of 4CL significantly increased in comparison to untreated plants (7- and 7.8-fold, respectively), progressively increasing to 8.31-fold at MeJA 150 μM, and subsequently decreasing to 6.49-fold (Figure 4B). *RAS* expression also increased significantly to 12-fold at MeJA 100 μM, peaked quickly at 17.1-fold with MeJA 150 μM, and then sharply decreased to 14.38-fold at MeJA 200 μM (Figure 4C).

As MeJA 10 μM was applied to the *S. abrotanoides* plants, the expression level of PAL greatly increased (6.37-fold) when compared to the control plant, but there was no discernible difference among MeJA treatments. MeJA 150 μM significantly increased *PAL* expression, which was 8.72-fold greater than in control plants (Figure 4A). After 24 h of treatment with MeJA 10 μM, 4CL significantly increased (5.51-fold), and with MeJA 100 μM, it increased even more, to 6.36-fold. At MeJA 150 and 200 μM, *4CL* expression started to decline after reaching its greatest level at MeJA 100 μM (6.36-fold), although it was still 4.51- and 4.37-fold greater than the level before treatment (Figure 4B). *RAS* gene expression in *S. abrotanoides* treated with MeJA rose at a rate of 6.38-fold at MeJA 10 μM, 7.78-fold at MeJA 100 μM, 10.87-fold at MeJA 150 μM, and 7.91-fold at MeJA 200 μM. The RA biosynthesis pathway, which includes *PAL* and *RAS*, had a consistent pattern of expression in both species as all gene transcript levels rose at all MeJA doses (Figure 4). The MeJA treatment increased the expression of the *PAL*, *4 CL*, and *RAS* genes so that their expression patterns were in accordance with the pattern of RA accumulation. Furthermore, the current study showed that spraying subgenus *Perovskia* species with high MeJA concentrations (200 μM) resulted in a decrease in the examined gene expression. These results are consistent with those found by Kianersi et al. [20,21,22,50], who found that large quantities of exogenously given MeJA suppressed expression. Examining the impact of external stimuli on the synthesis of secondary metabolites is useful for determining the highest performance of secondary metabolites and for clarifying their biosynthetic pathway(s) [76,77,78,79,80].

Both species showed similar patterns of *PAL* upregulation in response to MeJA therapy that paralleled those of RA accumulation. Likewise, a clear tread-off was observed between over-expression of the RA production-involved genes in several organs of *Ocimum basilicum* cultivars, lemon balm ecotypes, and *Agastache rugosa* [29,50,81]. Since PAL activity is rising in species belonging to the Lamiaceae family before RA accumulation [36,82], it has been hypothesized that it is a key enzyme for entrance into the phenylpropanoid pathway. Our research showed that *PAL* is essential for the synthesis of RA. Stress and plant species may affect the speed with which transcript levels rise and genes are induced. The expression profile of PAL in *Salvia miltiorrhiza* was analyzed, and it was shown to be upregulated in response to a wide variety of treatments [83,84]. Researchers have shown that activating MeJA increases PAL activity, a key enzyme in the phenylpro-panoid pathway [85,86]. Although our results unambiguously demonstrate the importance of *PAL* and *RAS* expression in RA generation in both species, the observed discrepancies in *PAL* and *RAS* expression as well as RA accumulation quantities are likely species-specific to the genus *Salvia*.

The strong connection between RA biosynthesis and RAS expression was highlighted by our findings. In both *Salvia* species treated with MeJA, there was a correlation between the pattern of RAS expression and the development of RA (Figure 2 and Figure 4). After treatment with MeJA 150 μM, *RAS* expression was 17.1 and 10.87 times higher in *S. yangii* and *S. abrotanoides*, respectively, compared to control plants. Similarly, the RA levels at this concentration were 1.66 times higher in *S. yangii* than in usual plants and 1.54 times higher in *S. abrotanoides*. The RAS in the studied species was reduced to 200 μM MeJA compared to 150 μM MeJA, and this was followed by a decrease in the synthesis of RA, phenol, and flavonoids. This gene may play a crucial role in regulating RA production because of the correlation between *RAS* expression and RA levels.

In general, our results were consistent with those of Kim et al. [55], who demonstrated that MeJA treatment enhanced the expression of the phenylpropanoid biosynthesis-related genes *ArPAL*, *Ar4CL*, and *ArC4H* in *A. rugosa*, ultimately leading to higher levels of RA accumulation. Plants exhibited rapid induction of PAL activity in response to MeJA elicitation, as reported by Mizukami et al. [62]. MeJA increases bioactive substance accumulation and changes the mRNA expression of genes involved in secondary metabolite manufacture in several plant species, as proven by numerous studies [46,47,48,76,77,87]. Similarly, Belhadj et al. [88] showed a rapid upregulation of the *PAL* gene expression in grape leaves using the MeJA treatment. Moreover, MeJA can alter CAD, PAL, and PPO activities as well as their relative mRNA levels [89].

Based on our results, MeJA at 150 μM was found to have the greatest levels of *PAL* and *RAS* expression, whereas control plants had the lowest levels, suggesting there is a strong association between amount of MeJA and RA. However, compared to the other dosages, leaves treated with MeJA at 100 μM generated the highest levels of the *4CL* transcript in the *S. abrotanoides* species. Finally, our results show that MeJA treatments directly regulated the accumulation of RA and total phenolic and flavonoid compounds, as well as the expression of key genes involved in RA biosynthesis (*PAL*, *4CL*, and *RAS*) in *Salvia* leaves.

## 3. Materials and Methods

### 3.1. Plant Materials and Environmental Factors

The seeds of *S. yangii* and *S. abrotanoides*, two species of *Salvia* subg. Perovskia kar, were cultivated in a glasshouse under controlled illumination (16 h day/8 h night, with a photosynthetic photon flux density of 310 mol m^−2^ s^−1^) and temperature (25/18 °C day/night) conditions.

### 3.2. MeJA Treatments

In the present study, *Salvia* plants were treated with MeJA at concentrations of 10, 100, 150, and 200 μM throughout their third month of development in a container. Distilled water served as a control (i.e., only possessing root, stem, and leaf components). The 0.22-m MILLIPORE pore-size filter membrane was used for full sterilisation of the MeJA (SIG-MA-ALDRICH) solutions. Final concentrations of MeJA and distilled water (control) were sprayed over the aerial tissues of two *Salvia* species until runoff. The experimental data were collected from three separate plants for each treatment. After the first day of treatment, both the control and treated leaves were sampled, frozen in liquid nitrogen, and maintained at −80 °C.

### 3.3. Real-Time PCR Analysis

Following the manufacturer’s instructions [20], total RNA was extracted from uniform young leaves of *Salvia* species, and cDNA was synthesized using the cDNA Synthesis Kit. As described before [56], we used gene-specific primers and the *actin* gene (as a housekeeping gene) to determine how varying amounts of MeJA affected the mRNA transcript levels of *PAL*, *4CL*, and *RAS* (Table 1). The fold-change (2^−ΔΔCt^) approach, which has been described earlier [90], was used for this investigation. Three technical and biological replicates were also employed for the gene expression study.

### 3.4. HPLC Analysis

To assess the impact of different MeJA concentrations on the formation of RA, Skendi et al.’s [91] approach was slightly modified. In a nutshell, 95 mg of the powdered dry leaf was mixed with 85% methanol (1:10 *w*/*v*), sonicated for 45 min, and then centrifuged at 3000 rpm for 15 min. The supernatant was mixed with sterile water to create the 20 mL reaction volume. To separate the RA, the resultant solution was filtered using a 0.45 M syringe filter before being injected onto an Agilent Technologies 1100 series HPLC system (C18 column (250 × 4.6 mm)). One milliliter of methanol/water (50/50 *v*/*v*) was produced with various concentrations of the RA standard, ranging from 1 to 350 mg/L. The calibration curve was created using the peak regions discovered from the injections. Mobile phase eluents A (acetonitrile) and B (water-acetic acid, 97.5:2.5, *v*/*v*) were used at a flow rate of 1 mL/min. The gradient of the solvent composition was 80A/20B for five minutes, followed by 50A/50B for ten minutes, and then 100% B for another fifteen minutes. Finally, the concentration of RA was determined as mg/g of dry weight after the RA molecule was detected at a wavelength of 330 nm (Figure S1A–C). Each sample was examined three times. Validation of the chromatographic peak of RA was conducted using the retention time of the reference standard. Agilent ChemStation software was used to measure the peak regions as part of the quantitative analysis with external standardization. The results were presented as mg/g DW.

### 3.5. Total Phenolic and Flavonoid Contents Analysis

**Figure 5:**
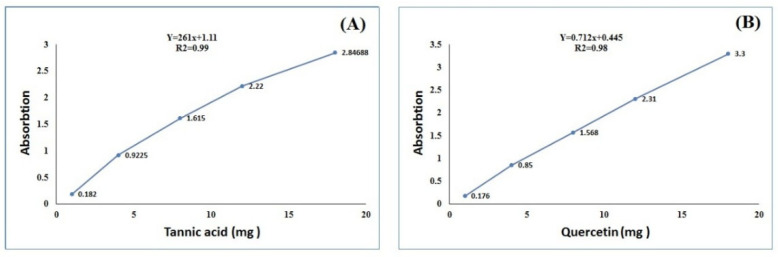
Quantitative analysis of total phenolic (**A**) and flavonoid content using a standard curve (**B**).

After being suspended in 80% methanol (25 mL) and shaken for 24 h at room temperature in a shaker, 1000 mg of dried and crushed *Salvia* leaf samples were turned into methanolic extracts to assess their total phenolic content (TPC) (150 rpm). The TPC was then calculated using the Folin–Ciocalteu reagent, as previously described [50], after the extracts had been filtered through two layers of Whatman paper. To begin, 2.5 mL of the Folin–Ciocalteu reagent (10-fold diluted) and 2 mL of sodium carbonate (7.5%) were added to 0.5 mL of the methanolic extract from each sample. The next step was to measure the absorbance at 765 nm after 15 min of heating at 45 °C. TPC was then determined as mg tannic acid equivalent/g dry weight (DW) (Figure 5A).

Additionally, Zhang et al.’s [92] chloride colorimetric technique was used to assess the total flavonoid content (TFC). First, 1.25 mL of water and 0.75 mL of sodium nitrate were mixed with 0.25 mg of each sample extract, and then the mixture was allowed to sit for 6 min in the dark. After 300 s of dark incubation, 0.15 mL of aluminum chloride (10%) was added to the liquid to complete the reaction. Each sample (5%) was then given 0.275 mL of water and 0.5 mL of sodium hydroxide solution. The TFC was shown as mg quercetin equivalent/g DW after reading the adsorption of the reaction solution at 510 nm (Figure 5B).

### 3.6. Data Analysis

The experimental data were analyzed using a factorial experiment based on a completely randomized design (CRD) with three replicates. The Duncan’s multiple range test was used to compare the means (DMRT). The statistical analysis was computed using SPSS ver. 16 software.

## 4. Conclusions

As the first report, our results revealed that exogenous MeJA treatment increases the contents of RA, TP, and TF, as well as the expression of key genes in the RA (phenylpropanoid) pathway such as *PAL*, *4CL*, and *RAS* in two species of *Salvia*. Hence, further research is required to understand how the accumulation of phenolic compounds in response to other treatments involving abiotic stresses interacts with the regulation of other genes engaged in this pathway. In the future, this process may be amenable to genetic modification in order to boost *Salvia’s* production of crucial chemicals.

## Figures and Tables

**Table 1:** Primers applied to real-time PCR analysis.

Real-Time Primers	Sequences (5′ to 3′)
PAL F PAL R	ACATCCTGGCCGTCCTATC GTCCTGCTCGTGCAGCTT
4CL F 4CL R	GCGATCTTGATCATGCAGAA AAGGTCATATTTGCCCACCA
RAS F RAS R	TCGATTTCTTGGAGCTGCAG GCACCCAACTAATCACCCAAAG
Actin Actin	ACCTCAAAATAGCATGGGGAAGT GGCCGTTCTCTCACTTTATGCTA

## Data Availability

Not applicable.

## Supplementary Materials

The following supporting information can be downloaded at: https://www.mdpi.com/article/10.3390/genes14040871/s1, Figure S1: Gas chromatogram for (A) Rosmarinic acid (RA) standard; (B) Rosmarinic acid (RA) product in control; and (C) RA product in 150 µM MeJA treated-plants.

## References

[1] Yilmaz A., Ciftci V. (2021). Genetic relationships and diversity analysis in Turkish laurel (*Laurus nobilis* L.) germplasm using ISSR and SCoT markers. Mol. Biol. Rep..

[2] Yilmaz A., Guler E., Soydemir H.E., Demirel S., Mollahaliloglu S., Karadeniz T., Ciftci V. (2021). Miracle. plant: Aronia (*Aronia melanocarpa*). MAS J. Appl. Sci..

[3] Yilmaz A., Karik Ü. (2022). AMF and PGPR enhance yield and secondary metabolite profile of basil (*Ocimum basilicum* L.). Ind. Crops Prod..

[4] Mátis A., Malkócs T., Kuhn T., Laczkó L., Moysiyenko I., Szabó A., Bădărău A., Sramkó G. (2023). Hiding in plain sight: Integrative analyses uncover a cryptic *Salvia* species in Europe. Taxon.

[5] Drew B.T., González-Gallegos J.G., Xiang C.L., Kriebel R., Drummond C.P., Walked J.B., Sytsma K.J. (2017). Salvia united: The greatest good for the greatest number. Taxon.

[6] Bielecka M., Pencakowski B., Stafiniak M., Jakubowski K., Rahimmalek M., Gharibi S., Matkowski A., Slusarczyk S. (2021). Metabolomics and DNA-based authentication of two traditional Asian medicinal and aromatic species of *Salvia* subg. Perovskia. Cells.

[7] Mohammadhosseini M., Venditti A., Akbarzadeh A. (2021). The genus *Perovskia* Kar.: Ethnobotany, chemotaxonomy and phytochemistry: A review. Toxin Rev..

[8] Ghaffari Z., Rahimmalek M., Sabzalian M.R. (2018). Variations in essential oil composition and antioxidant activity in *Perovskia abrotanoides* Kar. collected from different regions in Iran. Chem. Biodivers..

[9] Afshari M., Rahimmalek M. (2021). Variation in essential oil composition, anatomical, and antioxidant characteristics of *Achillea filipendulina* Lam. as affected by different phenological stages. J. Essent. Oil Res..

[10] Orhan E.I., Senol S.F., Ercetin T., Kahraman A., Celep F., Akaydin G., Sener B., Dogan M. (2013). Assessment of anticholinesterase and antioxidant properties of selected sage (*Salvia*) species with their total phenol and flavonoid contents. Ind. Crops Prod..

[11] Flora of Pakistan. http://www.efloras.org/flora_page.aspx?flora_id=5.

[12] Rechinger K.H. (1982). Flora Iranica.

[13] Mozaffarian V.A. (1996). Dictionary of Iranian Plant Names.

[14] Flora of China. http://www.efloras.org/flora_page.aspx?flora_id=2.

[15] Perveen S., Malik A., Noor A.T., Tareen R.B. (2008). Pervosides A and B, new isoferulyl glucosides from *Perovskia atriplicifolia*. J. Asian Nat. Prod. Res..

[16] Tareen R.B., Bibi T., Khan M.A., Ahmad M., Zafar M. (2010). Indigenous knowledge of folk medicine by the women of Kalat and Khuzdar regions of Balochistan, Pakistan. Pak. J. Bot..

[17] Baquar S.R. (1989). Medicinal and Poisonous Plants of Pakistan.

[18] Cardilea V., Russob A., Formisanoc C., Rigano D., Senatore F., Arnold N.A., Piozzi F. (2009). Essential oils of Salvia bracteata and Salvia rubifolia from Lebanon: Chemical composition, antimicrobial activity and inhibitory effect on human melanoma cells. J. Ethnol. Pharmacol..

[19] Eisenman S.W., Zaurov D.E., Struwe L. (2012). Medicinal Plants of Central Asia: Uzbekistan and Kyrgyzstan.

[20] Gao L., Zhou J., Zhu L.Y., Zhang J.R., Jing Y.X., Zhao J.W., Huang X.Z., Li G.P., Jiang Z.Y., Xue D.Y. (2017). Four New Diterpene Glucosides from *Perovskia atriplicifolia*. Chem. Biodivers..

[21] Jiang Z.Y., Yu Y.J., Huang C.G., Huang X.Z., Hu Q.F., Yang G.Y., Wang H.B., Zhang X.Y., Li G.P. (2015). Icetexane diterpenoids from *Perovskia atriplicifolia*. Planta Med..

[22] Kumar P.G., Gupta S., Murugan P.M., Bala Singh S. (2009). Ethnobotanical studies of Nubra valley—A cold arid zone of Himalaya. Ethnobot. Leafl..

[23] Moallem S.A., Niapour M. (2008). Study of embryotoxicity of *Perovskia abrotanoides*, an adulterant in folk-medicine, during organogenesis in mice. J. Ethnopharmacol..

[24] Ballabh B., Chaurasia O.P., Ahmed Z., Singh S.B. (2008). Traditional medicinal plants of cold desert Ladakh-Used against kidney and urinary disorders. J. Ethnopharmacol..

[25] Hosseinzadeh H., Amel S. (2009). Antinociceptive Effect of the Aerial Parts of *Perovskia abrotanoides* Extracts in Mice. Iran. Red Crescent Med. J..

[26] Sairafianpour M., Christensen J., Staerk D., Budnik B., Kharazmi A., Bagherzadeh K., Jaroszewski J.W. (2001). Leishmanicidal, antiplasmodial, and cytotoxic activity of novel diterpenoid 1,2-quinones from *Perovskia abrotanoides*: New source of tanshinones. J. Nat. Prod..

[27] Jaafari M.R., Hooshmand S., Samiei A., Hossainzadeh H. (2007). Evaluation of leishmanicidal effect of *Perovskia abrotanoides* Karel.Root extract by in vitro leishmanicidal assay using promastigotes of Leishmania major. Pharmacologyonline.

[28] Petersen M., Simmonds M.S.J. (2003). Molecules of interest: Rosmarinic acid. Phytochemistry.

[29] Hamaguchi T., Ono K., Murase A., Yamada M. (2009). Phenolic compounds prevent Alzheimer’s pathology through different effects on the amyloid-β aggregation pathway. Am. J. Pathol..

[30] Mazzanti G., Battinelli L., Pompeo C., Serrilli A.M., Rossi R., Sauzullo I., Megoni F., Vullo V. (2008). Inhibitory activity of *Melissa officinalis* L. extracts on Herpes simplex virus type 2 replication. Nat. Prod. Res..

[31] Al-Dhabi N.A., Arasu M.V., Park C.H., Park S.U. (2014). Recent studies on rosmarinic acid and its biological and pharmacological activities. EXCLI J..

[32] Taha R.A., Hassan M.M., Ibrahim E.A., Abo-Bakr N.H., Shaaban E.A. (2016). Carbon nanotubes impact on date palm in vitro cultures. Plant Cell Tissue Organ Cult..

[33] Petersen M. (2013). Rosmarinic acid: New aspects. Phytochem. Rev..

[34] Petersen M., Abdullah Y., Benner J., Eberle D., Gehlen K., Hücherig S., Janiak V., Kim K.H., Sander M., Weitzel C. (2009). Evolution of rosmarinic acid biosynthesis. Phytochemistry.

[35] Weitzel C., Petersen M. (2011). Cloning and characterization of rosmarinic acid synthase from *Melissa officinalis* L.. Phytochemistry.

[36] Deng C., Wang Y., Huang F., Lu S., Zhao L., Ma X., Kai G. (2020). SmMYB2 promotes salvianolic acid biosynthesis in the medicinal herb *Salvia miltiorrhiza*. J. Integr. Plant. Biol..

[37] Zhang S., Li H., Liang X., Yan Y., Xia P., Jia Y., Liang Z. (2015). Enhanced production of phenolic acids in *Salvia miltiorrhiza* hairy root cultures by combing the RNAi-mediated silencing of chalcone synthase gene with salicylic acid treatment. Biochem. Eng. J..

[38] Fu R., Shi M., Deng C., Zhang Y., Zhang X., Wang Y., Kai G. (2020). Improved phenolic acid content and bioactivities of *Salvia miltiorrhiza* hairy roots by genetic manipulation of RAS and CYP98A14. Food Chem..

[39] Guerriero G., Berni R., Muñoz-Sanchez J.A., Apone F., Abdel-Salam E.M., Qahtan A.A., Alatar A.A., Cantini C., Cai G., Hausman J.-F. (2018). Production of plant secondary metabolites: Examples, tips and suggestions for biotechnologists. Genes.

[40] Naik P.M., Al–Khayri J.M., Shanker A.K., Shanker C. (2016). Abiotic and Biotic Elicitors—Role in Secondary Metabolites Production through In Vitro Culture of Medicinal Plants. Abiotic and Biotic Stress in Plants—Recent Advances and Future Perspectives.

[41] Gonçalves S., Romano A., Vijayakumar R., Raja S.S.S. (2018). Production of Plant Secondary Metabolites by Using Biotechnological Tools. Secondary Metabolites: Sources and Applications.

[42] Sircar D., Cardoso H.G., Mukherjee C., Mitra A., Arnholdt-Schmitt B. (2012). Alternative oxidase (AOX) and phenolic metabolism in methyl jasmonate-treated hairy root cultures of *Daucus carota* L.. J. Plant Physiol..

[43] Narayani M., Srivastava S. (2017). Elicitation: A stimulation of stress in in vitro plant cell/tissue cultures for enhancement of secondary metabolite production. Phytochem. Rev..

[44] Gai Q., Jiao J., Wang X., Zang Y., Niu L., Fu Y. (2019). Elicitation of *Isatis tinctoria* L. hairy root cultures by salicylic acid and methyl jasmonate for the enhanced production of pharmacologically active alkaloids and flavonoids. Plant Cell Tissue Organ Cult..

[45] Xing B., Yang D., Liu L., Han R., Sun Y., Liang Z. (2018). Phenolic acid production is more effectively enhanced than tanshinone production by methyl jasmonate in *Salvia miltiorrhiza* hairy roots. Plant Cell Tissue Organ Cult..

[46] Fatemi F., Abdollahi M.R., Mirzaie-asl A., Dastan D., Garagounis C., Papadopoulou K. (2019). Identification and expression profiling of rosmarinic acid biosynthetic genes from *Satureja khuzistanica* under carbon nanotubes and methyl jasmonate elicitation. Plant Cell Tissue Organ Cult..

[47] Kianersi F., Abdollahi M.R., Mirzaie-asl A., Dastan D., Rasheed F. (2020). Identification and tissue-specific expression of rutin biosynthetic pathway genes in *Capparis spinosa* elicited with salicylic acid and methyl jasmonate. Sci. Rep..

[48] Kianersi F., Abdollahi M.R., Mirzaie-asl A., Dastan D., Rasheed F. (2020). Biosynthesis of rutin changes in *Capparis spinosa* due to altered expression of its pathway genes under elicitors’ supplementation. Plant Cell Tissue Organ Cult..

[49] Kianersi F., PourAboughadareh A., Majdi M., Poczai P. (2021). Effect of Methyl Jasmonate on Thymol, Carvacrol, Phytochemical Accumulation, and Expression of Key Genes Involved in Thymol/Carvacrol Biosynthetic Pathway in Some Iranian Thyme Species. Int. J. Mol. Sci..

[50] Kianersi F., Amin Azarm D., Pour-Aboughadareh A., Poczai P. (2022). Change in Secondary Metabolites and Expression Pattern of Key Rosmarinic Acid Related Genes in Iranian Lemon Balm (*Melissa officinalis* L.) Ecotypes Using Methyl Jasmonate Treatments. Molecules.

[51] Abdollahi M.R., Kianersi F., Moosavi S.S., Dastan D., Asadi S. (2022). Identification and Expression Analysis of Two Genes Involved in the Biosynthesis of t-Anethole in Fennel (*Foeniculum vulgare* Mill.) and Their Up-Regulation in Leaves in Response to Methyl Jasmonate Treatments. J. Plant Growth Regul..

[52] Kianersi F., Amin Azarm D., Fatemi F., Pour-Aboughadareh A., Poczai P. (2022). Methyl Jasmonate Induces Genes Involved in Linalool Accumulation and Increases the Content of Phenolics in Two Iranian Coriander (*Coriandrum sativum* L.) Ecotypes. Genes.

[53] Zhang S., Yan Y., Wang B., Liang Z., Liu Y., Liu F., Qi Z. (2014). Selective responses of enzymes in the two parallel pathways of rosmarinic acid biosynthetic pathway to elicitors in *Salvia miltiorrhiza* hairy root cultures. J. Biosci. Bioeng..

[54] Yousefian S., Lohrasebi T., Farhadpour M., Haghbeen K. (2020). Effect of methyl jasmonate on phenolic acids accumulation and the expression profile of their biosynthesis-related genes in *Mentha spicata* hairy root cultures. Plant Cell Tissue Organ Cult..

[55] Kim Y.B., Kim J.K., Uddin M.R., Xu H., Park W.T., Tuan P.A., Li X., Chung E.S., Lee J.-H., Park S.U. (2013). Metabolomics analysis and biosynthesis of rosmarinic acid in *Agastache rugosa* Kuntze treated with methyl jasmonate. PLoS ONE.

[56] Stafiniak M., Ślusarczyk S., Pencakowski B., Matkowski A., Rahimmalek M., Bielecka M. (2021). Seasonal Variations of Rosmarinic Acid and Its Glucoside and Expression of Genes Related to Their Biosynthesis in Two Medicinal and Aromatic Species of *Salvia* subg. Perovskia. Biology.

[57] Fatemi F., Abdollahi M.R., Mirzaie-Asl A., Dastan D., Papadopoulou K. (2020). Phytochemical, antioxidant, enzyme activity and antifungal properties of *Satureja khuzistanica* in vitro and in vivo explants stimulated by some chemical elicitors. Pharm. Biol..

[58] Ruan J., Zhou Y., Zhou M., Yan J., Khurshid M., Weng W., Cheng J., Zhang K. (2019). Jasmonic acid signaling pathway in plants. Int. J. Mol. Sci..

[59] Xiao Y., Gao S., Di P., Chen J., Chen W., Zhang L. (2009). Methyl jasmonate dramatically enhances the accumulation of phenolic acids in *Salvia miltiorrhiza* hairy root cultures. Physiol. Plant..

[60] Park W.T., Arasu M.V., Al-Dhabi N.A., Yeo S.K., Jeon J., Park J.S., Lee S.Y., Park S.U. (2016). Yeast extract and silver nitrate induce the expression of phenylpropanoid biosynthetic genes and induce the accumulation of rosmarinic acid in *Agastache rugosa* cell culture. Molecules.

[61] Kintzios S., Makri O., Panagiotopoulos E., Scapeti M. (2003). In vitro rosmarinic acid accumulation in sweet basil (*Ocimum basilicum* L.). Biotechnol. Lett..

[62] Mizukami H., Tabira Y., Ellis B.E. (1993). Methyl jasmonate-induced rosmarinic acid biosynthesis in *Lithospermum erythrorhizon* cell suspension cultures. Plant. Cell. Rep..

[63] Guan Y., Hu W., Jiang A., Xu Y., Sa R., Feng K., Zhao M., Yu J., Ji Y., Hou M. (2019). Effect of Methyl Jasmonate on Phenolic Accumulation in Wounded Broccoli. Molecules.

[64] Yamamoto R., Ma G., Zhang L., Hirai M., Yahata M., Yamawaki K., Shimada T., Fujii H., Endo T., Kato M. (2020). Effects of Salicylic Acid and Methyl Jasmonate Treatments on Flavonoid and Carotenoid Accumulation in the Juice Sacs of *Satsuma Mandarin* In Vitro. Appl. Sci..

[65] Salami M., Rahimmalek M., Ehtemam M.H. (2016). Inhibitory effect of different fennel (*Foeniculum vulgare*) samples and their phenolic compounds on formation of advanced glycation products and comparison of antimicrobial and antioxidant activities. Food Chem..

[66] Roby M.H.H., Sarhana M.A., Selima K.A., Khalela K.I. (2013). Antioxidant and Antimicrobial Activities of Essential Oil and Extracts of Fennel (*Foeniculum vulgare* L.) and Chamomile (*Matricaria chamomilla* L.). Ind. Crops Prod..

[67] Kim H.J., Chen F., Wang X., Choi J.H. (2006). Effect of methyl jasmonate on phenolics, isothiocyanate, and metabolic enzymes in radish sprout (*Raphanus sativus* L.). J. Agric. Food. Chem..

[68] Kim H.J., Chen F., Wang X., Rajapakse N.C. (2006). Effect of methyl jasmonate on secondary metabolites of sweet basil (*Ocimum basilicum* L.). J. Agric. Food. Chem..

[69] Kim H.J., Park K.J., Lim J.H. (2011). Metabolomic analysis of phenolic compounds in buckwheat (*Fagopyrum esculentum* M.) sprouts treated with methyl jasmonate. J. Agric. Food. Chem..

[70] Awasthi P., Mahajan V., Jamwal V.L., Chouhan R., Kapoor N., Bedi Y.S., Gandhi S.G. (2019). Characterization of the gene encoding 4-coumarate: CoA ligase in *Coleus forskohlii*. J. Plant. Biochem. Biotechnol..

[71] Deng Y., Li C., Li H., Lu S., Iriti M. (2018). Identification and characterization of flavonoid biosynthetic enzyme genes in *Salvia miltiorrhiza* (Lamiaceae). Molecules.

[72] Park C.H., Yeo H.J., Park Y.E., Chun S.W., Chung Y.S., Lee S.Y., Park S.U. (2019). Influence of Chitosan, Salicylic Acid and Jasmonic Acid on Phenylpropanoid Accumulation in Germinated Buckwheat (*Fagopyrum esculentum* Moench). Foods.

[73] Jaafar H.Z., Ibrahim M.H., Mohamad-Fakri N.F. (2012). Impact of soil field water capacity on secondary metabolites, phenylalanine ammonia-lyase (PAL), maliondialdehyde (MDA) and photosynthetic responses of Malaysian Kacip Fatimah (*Labisia pumila* Benth). Molecules.

[74] Zhou P., Li Q., Liu G., Xu N., Yang Y., Zeng W., Chen A., Wang S. (2018). Integrated analysis of transcriptomic and metabolomic data reveals critical metabolic pathways involved in polyphenol biosynthesis in *Nicotiana tabacum* under chilling stress. Funct. Plant Biol..

[75] Gharibi S., Sayed Tabatabaei B.E., Saeidi G., Talebi M., Matkowski A. (2019). The effect of drought stress on polyphenolic compounds and expression of flavonoid biosynthesis related genes in *Achillea pachycephala* Rech.f. Phytochemistry.

[76] Majdi M., Abdollahi M.R., Maroufi A. (2015). Parthenolide accumulation and expression of genes related to parthenolide biosynthesis affected by exogenous application of methyl jasmonate and salicylic acid in *Tanacetum parthenium*. Plant. Cell. Rep..

[77] Elyasi R., Majdi M., Bahramnejad B., Mirzaghaderi G. (2016). Spatial modulation and abiotic elicitors responses of the biosynthesis related genes of mono/triterpenes in black cumin (*Nigella sativa*). Ind. Crops Prod..

[78] Brouki Milan E., Mandoulakani B.A., Kheradmand F. (2017). The effect of methyl jasmonate on the expression of phenylalanine ammonia lyase and eugenol-o-methyl transferase genes in basil. Philipp. Agric. Sci..

[79] Chezem W.R., Clay N.K. (2016). Regulation of plant secondary metabolism and associated specialized cell development by MYBs and bHLHs. Phytochemistry.

[80] Açıkgoz M. (2020). Establishment of cell suspension cultures of *Ocimum basilicum* L. and enhanced production of pharmaceutical active ingredients. Ind. Crops Prod..

[81] Li X., Kim J.K., Park S.U. (2017). Molecular cloning and characterization of rosmarinic acid biosynthetic genes and rosmarinic acid accumulation in *Ocimum basilicum* L.. Saudi. J. Biol. Sci..

[82] Thiyagarajan K., Vitali F., Tolaini V., Galeffi P., Cantale C., Vikram P., Singh S., De Rossi P., Nobili C., Procacci S. (2016). Genomic characterization of phenylalanine ammonia lyase gene in buckwheat. PLoS ONE.

[83] Hao X., Pu Z., Cao G., You D., Zhou Y., Deng C., Shi M., Nile S.H., Wang Y., Zhou W. (2020). Tanshinone and salvianolic acid biosynthesis are regulated bySmMYB98 in *Salvia miltiorrhiza* hairy roots. J. Adv. Res..

[84] Huang J., Gu M., Lai Z., Fan B., Shi K., Zhou Y.H., Yu J.Q., Chen Z. (2010). Functional analysis of the Arabidopsis PAL gene family in plant growth, development, and response to environmental stress. Plant Physiol..

[85] Cocetta G., Rossoni M., Gardana C., Mignani I., Ferrante A., Spinardi A. (2015). Methyl jasmonate affects phenolic metabolism and gene expression in blueberry (*Vaccinium corymbosum*). Physiol. Plant..

[86] Biswas T. (2020). Elicitor induced increased rosmarinic acid content of in vitro root cultures of *Ocimum basilicum* L. (Sweet Basil). Plant Sci. Today.

[87] Sarabandi M., Farokhzad A., Mandoulakani B.A., Ghasemzadeh R. (2019). Biochemical and gene expression responses of two Iranian grape cultivars to foliar application of methyl jasmonate under boron toxicity conditions. Sci. Hort..

[88] Belhadj A., Saigne C., Telef N., Cluzet S., Bouscaut J., Corio-Costet M.F., Mérillon J.M. (2006). Methyl jasmonate induces defense responses in grapevine and triggers protection against Erysiphe necator. J. Agric. Food Chem..

[89] Farooq M.A., Gill R.A., Islam F., Ali B., Liu H., Xu J., He S., Zhou W. (2016). Methyl jasmonate regulates antioxidant defense and suppresses arsenic uptake in *Brassica napus* L.. Front. Plant Sci..

[90] Livak K.J., Schmittgen T.D. (2001). Analysis of relative gene expression data using real-time quantitative PCR and the 2^−ΔΔct^ method. Methods.

[91] Skendi A., Irakli M., Chatzopoulou P. (2017). Analysis of phenolic compounds in Greek plants of Lamiaceae family by HPLC. J. Appl. Res. Med. Aromat. Plants.

[92] Zhang D.Y., Yao X.H., Duan M.H., Wei F.Y., Wu G.H., Li L. (2015). Variation of essential oil content and antioxidant activity of Lonicera species in different sites of China. Ind. Crops Prod..

