# Supplementary material for: The Influence of Methyl Jasmonate on Expression Patterns of Rosmarinic Acid Biosynthesis Genes, and Phenolic Compounds in Different Species of Salvia subg. Perovskia Kar L
Source: Genes (Basel). 2023 Apr 5;14(4):871. doi: 10.3390/genes14040871 (PMC10137496; doi:10.3390/genes14040871)
Supplement: Supplementary file 1 [file genes-14-00871-s001.zip › genes-2247474-supplementary.pdf]

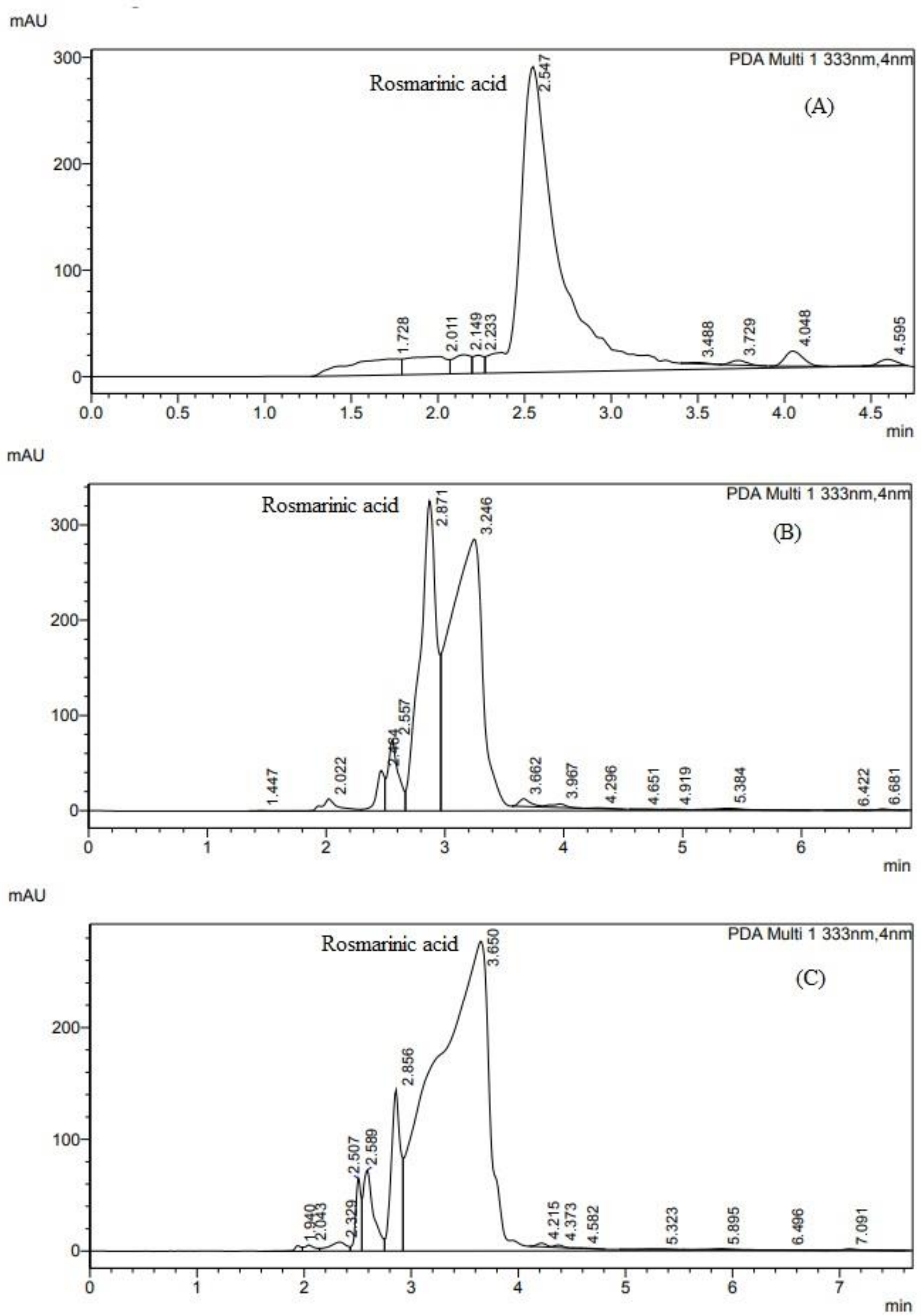

Figure S1. Gas chromatogram for (A) Rosmarinic acid (RA) standard (B) Rosmarinic acid (RA) product in control and (C) RA product in 150 µM MeJA treated-plants.
